# The reliability and discriminant validity of physical, technical, and perceptual-physiological measures during a game-specific basketball activity simulation protocol

**DOI:** 10.3389/fpsyg.2024.1414339

**Published:** 2024-06-21

**Authors:** Davide Ferioli, Pedro E. Alcaraz, Tomás T. Freitas, Fabio Trimarchi, Daniele Conte, Lorenzo Formica, Linda H. Chung, Aaron T. Scanlan

**Affiliations:** ^1^Department of Biomedical Sciences, Dental Sciences, and Morpho-Functional Imaging, University of Messina, Messina, Italy; ^2^UCAM Research Center for High Performance Sport, UCAM Universidad Católica de Murcia, Murcia, Spain; ^3^SCS—Strength & Conditioning Society, Murcia, Spain; ^4^Facultad de Deporte, UCAM Universidad Católica de Murcia, Murcia, Spain; ^5^NAR—Nucleus of High Performance in Sport, São Paulo, Brazil; ^6^Department of Movement, Human and Health Sciences, University of Rome “Foro Italico”, Rome, Italy; ^7^School of Health, Medical, and Applied Sciences, Central Queensland University, Rockhampton, QLD, Australia

**Keywords:** team sport, testing, retest, shooting, RPE, heart rate, simulated match-play

## Abstract

Activity simulation protocols offer useful applications in research and practice; however, the specificity of such protocols to basketball game-play is currently lacking. Consequently, this study aimed to develop a game-specific basketball activity simulation protocol representative of typical playing durations and assess its reliability and discriminant validity. The simulation protocol was modified from an original version (i.e., Basketball Exercise Simulation Test) to incorporate regular breaks indicative of time-outs, free-throws, and substitutions. Twelve competitive male and female adult basketball players competing in the fourth or fifth Spanish basketball division underwent repeated trials of the simulation protocol (min. 4 to max. 14 days apart) for reliability analyses. In turn, 13 competitive male (fifth division), 9 competitive female (fourth division), and 13 recreational male adult basketball players completed the simulation protocol to assess discriminant validity via comparisons between sexes (competitive players) and playing levels (males). A range of physical, technical, and perceptual-physiological variables were collected during and following the simulation protocol. Several physical and heart rate variables displayed the strongest reliability (intraclass correlation coefficient [ICC] = 0.72–0.96; coefficient of variation [CV] = 1.78–6.75%), with physical decrement, technical, blood lactate concentration, and rating of perceived exertion (RPE) variables having the weakest (ICC = 0.52–0.75; CV = 10.34–30.85%). Regarding discriminant analyses between sexes, males demonstrated significantly greater physical outputs in several variables and lower RPE compared to females (*p* < 0.05, *moderate*-to-*large* effects). Comparisons between playing levels revealed competitive males had significantly greater physical outputs across many variables, alongside higher mean heart rate and lower RPE than recreational males (*p* < 0.05, *moderate*-to-*large* effects). This study presents a novel game-specific basketball activity simulation protocol replicating actual playing durations and game configurations that might be successfully applied for both training and research purposes. Reliability statistics are provided for several variables to inform end-users on potential measurement error when implementing the simulation protocol. Discriminant validity of the simulation protocol was supported for several variables, suggesting it may hold practical utility in benchmarking or selecting players. Future research on this topic is encouraged examining wider samples of male and female basketball players at different levels as well as additional forms of validity for the protocol.

## 1 Introduction

Basketball is one of the most popular global sports across different age groups and sexes with numerous competitions played at different levels in several countries (Ferioli et al., 2022). At higher playing levels, such as in semi-professional, professional, and representative contexts, teams are more likely to hire dedicated interdisciplinary support staff given their increased budgets and emphasis on performance-driven outcomes (Gleason et al., 2024a). The support staff predominantly aims to optimize player health and performance through observation, analysis, and management of players, as well as input in selection, development, training, and recovery practices (Gleason et al., 2024b). A crucial initial step in fulfilling these functions is acquiring a thorough understanding of the physical, technical, and perceptual-physiological competitive requirements the players face in their specific context (Russell et al., 2021). This knowledge then permits highly specific player training and management strategies to be developed within teams.

To ensure player plans are progressing as intended, controlled assessments with desired physical, technical, and perceptual-physiological measures must be taken periodically. In this regard, in-game measurements do not represent standardized stimuli to accurately assess changes in players across time due to the stochastic nature of game demands which are related to various contextual factors (e.g., opponent quality, team tactics, scoreline, and individual playing time) (Stojanović et al., 2018). Moreover, it is often difficult to take measurements on players during basketball competition given that some leagues prohibit the use of popularized wearable technologies that capture useful physical (e.g., microsensors) and physiological measures (e.g., heart rate monitors). Consequently, simulation protocols are a viable option to gather various measures on players in a controlled manner during game-specific activity bouts outside of a competition context (Williams et al., 2010; Waldron et al., 2013). In this way, physical (Fox et al., 2017), technical (Boddington et al., 2019), and perceptual-physiological (Lupo et al., 2017; Berkelmans et al., 2018) variables are regularly assessed among basketball players in the literature and shown to be of interest to end-users working with teams (Fox et al., 2020).

To date, various basketball-specific simulation protocols have been developed (Kostopoulos et al., 2004; Afman et al., 2014); however, the Basketball Exercise Simulation Test has been the most popular basketball-specific simulation protocol adopted within the literature (Scanlan et al., 2012, 2014, 2017a,b, 2018; Staunton et al., 2017; Delextrat et al., 2018; Latzel et al., 2018; Hovsepian et al., 2021; Javanmardi et al., 2021; Bourdas et al., 2024). This test replicates the intermittent activity profile and distances measured during games in professional, male basketball players competing in the Australian National Basketball League (Scanlan et al., 2014). In turn, it has been used to comprehensively quantify the demands of game-specific basketball activity (Latzel et al., 2018; Scanlan et al., 2018), assess the efficacy of nutritional (Delextrat et al., 2018) and training interventions (Hovsepian et al., 2021), as a training strategy (Javanmardi et al., 2021), as a fatiguing protocol (Bourdas et al., 2024), and to assess monitoring approaches (Staunton et al., 2017; Scanlan et al., 2017a,b). The original version of the test was developed to simulate the maximum demands likely encountered during games (i.e., 48 min across 4 × 12-min quarters of live playing time) with reported reliability statistics (Scanlan et al., 2014) and various types of supported validity (Scanlan et al., 2012, 2014). However, players are not likely to compete for entire games due to team substitution strategies and the original version of the test does not account for the frequent breaks encountered during games (e.g., free-throws, time-outs), reducing its applicability to actual competitive requirements. In support of this notion, the test has since been modified with reduced activity durations in some studies (Staunton et al., 2017; Delextrat et al., 2018; Latzel et al., 2018).

Despite the need for an adapted, game-specific basketball simulation, no dedicated research has proposed an alternative protocol nor assessed its reliability and validity. Establishing the retest reliability of test protocols is essential to ensure they can suitably detect changes in outcomes (Weakley et al., 2023). Moreover, while many types of validity exist, discriminant validity is useful as it is predicated on the premise that test outcomes are unrelated between different groups (Weakley et al., 2023). With acceptable retest reliability and discriminant validity, end-users can apply testing protocols confidently for longitudinal monitoring and distinguishing between performance levels in practice. Therefore, the aims of this study were to: (1) develop a new game-specific basketball activity simulation protocol representative of typical playing durations; and (2) assess the reliability and discriminant validity of this protocol.

## 2 Materials and methods

All data were collected across June and July in 2022, which was within 2 months of finishing the 2021–2022 Primera Nacional Spanish basketball competition for competitive players. All players were familiarized with testing procedures before official data collection began via demonstration, observation, and trials of the protocol as used previously (Scanlan et al., 2012, 2014). For reliability analyses, a repeated-measures, within-subject design was followed whereby players completed the simulation protocol on two separate occasions, with a minimum of 4 days and a maximum of 14 days between trials. While testing time was randomly allocated to players, each player was assessed at the same time of day in each trial (when completing repeated trials) to avoid any circadian variations in physical performance as documented previously in basketball players (Gaos et al., 2023). For discriminant validity analyses, a cross-sectional, between-subjects design was followed whereby players were only required to complete the simulation protocol on a single occasion. During the testing period, all players were instructed to maintain regular nutritional and sleeping behaviors and to abstain from physical activity for 24 h before each testing trial – which was verbally confirmed with each player prior to testing. All testing sessions were performed on the same indoor basketball court in a controlled air-conditioned environment.

### 2.1 Participants

Different player samples were included in the reliability and validity analyses within this study. Firstly, 12 adult basketball players (males: *n* = 8; age: 24.1 ± 4.2 years; stature: 185 ± 9 cm; body mass: 84.9 ± 16.7 kg; females: *n* = 4; age: 24.3 ± 2.4 years; stature: 169 ± 9 cm; body mass: 63.3 ± 7.6 kg) were recruited for the reliability analyses. Secondly, 35 adult basketball players (competitive males: *n* = 13; age: 25.2 ± 4.0 years; stature: 185 ± 9 cm; body mass: 85.9 ± 14.0 kg; competitive females: *n* = 9; age: 21.4 ± 2.9 years; stature: 170 ± 9 cm; body mass: 64.3 ± 6.6 kg; recreational males: *n* = 13; age: 29.2 ± 7.6 years; stature: 184 ± 7 cm; body mass: 87.9 ± 24.0 kg) were recruited for discriminant validity analyses between sexes (i.e., competitive males vs. competitive females) and playing levels (i.e., competitive males vs. recreational males). *A priori* power analysis (G*Power, version 3.1.9.7; University of Düsseldorf, Germany) indicated a minimum of 8 players was needed using an *α* = 0.05, *β* = 0.95, and effect size = 1.97, based on research examining a similar protocol for one of the main variables (i.e., mean circuit time) (Scanlan et al., 2012). All competitive male players were competing in the fifth division of the Spanish basketball competition, while all females were competing in the fourth division of the Spanish basketball competition. Recreational male players were regularly participating in non-structured basketball activity. Players competing in the Spanish basketball competitions performed at least three on-court team training sessions (each ~90–120 min) and a game per week during the season. It should be noted that data from the first trial in all players completing the reliability testing were included in both discriminant validity analyses, and data from competitive male players in discriminant validity analyses between sexes were also included in analyses between playing levels. Players were of various nationalities and volunteered to participate after being informed of the study procedures, risks, and benefits. For inclusion, players had to be adults (≥18 years of age) participating in the fourth or fifth division of the Spanish basketball competition or recreational basketball, and be healthy with no injuries across the study. All procedures were approved by the UCAM Universidad Católica de Murcia’s Ethics Committee with written informed consent obtained from each player prior to participation.

### 2.2 Procedures

#### 2.2.1 Basketball activity simulation protocol

**Figure 1:**
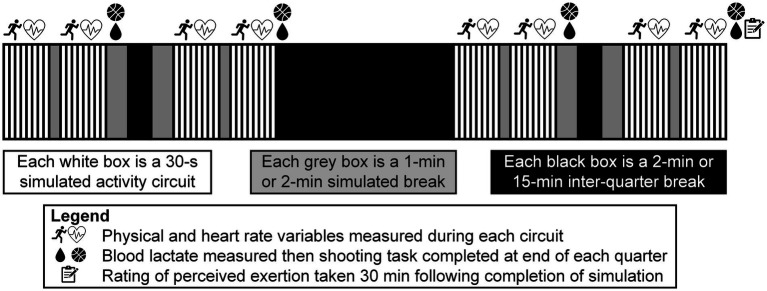
Configuration of the game-specific basketball activity simulation protocol.

**Figure 2:**
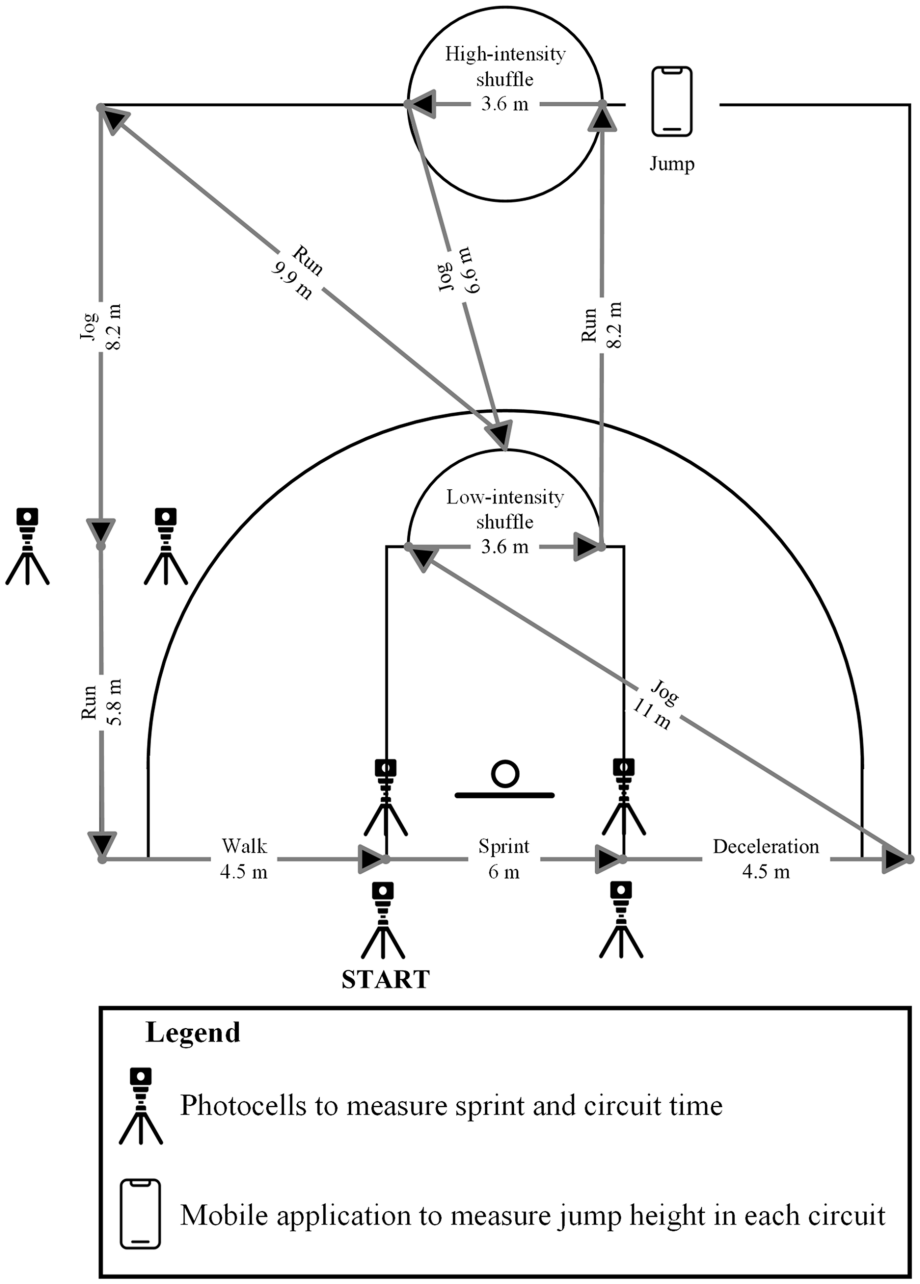
The activity breakdown within each circuit of the game-specific basketball activity simulation protocol.

A standardized 15-min warm-up was performed before each trial and consisted of 5 min of active mobility exercises, 5 min of running skill and basketball specific skill exercises, ten 2-point shots, ten 3-point shots, and ten free-throws. Players then underwent familiarization (i.e., receiving verbal instructions and completing two circuit trials) before completing the simulation protocol. The protocol lasted a total of 63 min with 32 min of activity being performed, representing live active play. This configuration was chosen to reflect the typical playing time among players competing in Spanish basketball competition as indicated by past research (Lopez-Laval et al., 2016) and official competition statistics^1^ (opposed to the 48 min of activity included in the original version of the protocol (Scanlan et al., 2012)) along with the likely occurrence of in-game stoppages (i.e., time-outs, free-throws, and inter-quarter breaks). The simulation protocol was split into quarters, with each quarter involving two 4-min activity bouts separated by 1-min of passive seated rest (corresponding to a time-out duration), and a 2-min passive seated rest (corresponding to a substitution). Each quarter was further separated by a 2-min passive seated rest, with a 15-min passive seated rest between the second and third quarters (i.e., half-time break) in line with international regulations. This protocol configuration is shown in Figure 1. Each 4-min simulated activity bout consisted of eight 30-s circuits at guided intensities that were self-regulated. These circuits were arranged identically to those stipulated for the original version of the simulation protocol (Scanlan et al., 2012). Each activity performed in the simulation protocol were described to players before testing to guide movement intensities and included: walking – activity at no greater intensity than walking pace; jogging – activity at a moderate intensity, higher than walking pace but without urgency (50% of maximal velocity); running – activity at a greater than moderate intensity, with effort and purpose but still below maximal exertion (75% of maximal velocity); sprinting – all-out effort at maximal intensity; low-intensity shuffling – activity characterized by shuffling action of the feet within a defensive stance position, performed without urgency; high-intensity shuffling – activity characterized by shuffling action of the feet within a defensive stance position, performed at maximal effort; and jumping – countermovement maximal effort jump initiated off both legs with arm swing. The breakdown of activities in each circuit of the simulation protocol is displayed in Figure 2.

To ensure players did not initiate each circuit with momentum, they were required to start each circuit in a stationary position 30 cm behind the initial set of timing lights via floor markings. Each circuit lasted for 30 s (maximum of 16 circuits completed per 8 min of activity per quarter). If players completed the circuit in under 30 s, remaining time was used as passive standing rest at the starting point. If players took longer than 30 s to complete the circuit, they were required to completely stop then immediately commence the following circuit. In these cases, players completed less than 8 circuits per 4-min bout unless adequate timing was restored (i.e., they were completing circuits within 30 s when averaged across the 4-min bout). Players were given standardized verbal instructions and encouragement to ensure correct execution and optimal performance were obtained. A range of variables were collected for each player during testing and tabulated across the entire simulation protocol rather than reported per quarter.

#### 2.2.2 Physical variables

Firstbeat Sports sensors (Firstbeat Technologies Oy; Jyväskylä, Finland) were used to measure movement load continuously for each player when completing the circuits during the simulation protocol (i.e., with data recorded during breaks trimmed). Players wore sensors firmly affixed to their chest roughly at the base of the sternum via textile straps. The same sensor was worn by all players across trials to avoid any inter-sensor variations in data outputs. Movement load (in arbitrary units [AU]) was calculated via Firstbeat Sports software (version 2.50.3; Firstbeat Technologies Oy; Jyväskylä, Finland) as the sum of accelerations across the three movement axes using the tri-axial accelerometer component sampling at 50 Hz with the following formula:


$$\mathrm{Movement}\;\mathrm{load}=\sqrt{\frac{{\left({Α}_{y1}-{Α}_{y-1}\right)}^{2}+{\left({Α}_{x1}-{Α}_{x-1}\right)}^{2}+{\left({Α}_{z1}-{Α}_{z-1}\right)}^{2}}{300}}$$

where A_y_, A_x_, and A_z_ are the orthogonal components measured from the triaxial accelerometer. Movement intensity was calculated as relative movement load per minute (AU . min^−1^) of activity during the simulation protocol (i.e., removing any breaks). Data were exported into Microsoft Excel (version 2,402; Microsoft Corporation; Redmond, WA, USA) for processing following testing. Distance, time, height, and decrement outcomes were also recorded as per previous methodologies (Scanlan et al., 2012, 2018). Distance covered during the simulation protocol was calculated following each test by marking the precise end-point upon completion (if all allotted circuits were not completed) and measuring the distance covered in this final circuit with a measuring tape. It should be noted that if players were able to complete all circuits in a 4-min bout, the distance of the final circuit ceased at the photocells following the jog. If players completed all allotted circuits across all quarters, they covered a total distance of 4519.2 m (564.9 m per 4-min bout consisting of 7 circuits x 71.9 m and 1 circuit x 61.6 m). Performance times to complete each sprint (per circuit) and each circuit (measured following the jog) were measured using single-beam photocells (Witty gate; Microgate; Bolzano, Italy) set at ~1.1 m above ground level. While the reliability and validity of these specific photocells are yet to be investigated, this technology has been shown to be reliable previously (Thapa et al., 2023). Jump height for each jump (per circuit) was recorded using an iPhone 13 high-speed camera (Apple, California, USA) and analyzed using a valid and reliable mobile application (Gencoglu et al., 2023) (My Jump 2). Due to a technical error, jump data were not collected for recreational players and therefore excluded from discriminative validity analyses between playing levels. Sprint, circuit, and jump decrements were determined as the cumulative percent decline using the mean outcome across each two sequential circuits inputted into the following formulae:


$$\begin{array}{l} \mathrm{Sprint}/\mathrm{circuit}\;\mathrm{decrement}\;\left(\%\right)=\\ \left(\left[\mathrm{total}\;\mathrm{time}/\mathrm{ideal}\;\mathrm{time}\right]\times 100\right)-100 \end{array}$$

$$\begin{array}{l} \mathrm{Jump}\;\mathrm{decrement}\;\left(\%\right)=\\ 100-\left(\left[\mathrm{total}\;\mathrm{jump}\;\mathrm{height}/\mathrm{ideal}\;\mathrm{jump}\;\mathrm{heigth}\right]\times 100\right) \end{array}$$

where total values were the sum of all two-circuit mean outcomes and ideal values were the best sequential two-circuit outcome.

#### 2.2.3 Technical variables

Players performed a shooting task for technical assessment following perceptual-physiological measures being taken at the end of each quarter. The shooting task consisted of shooting 25 consecutive free-throws (4.6 m from directly below the backboard) on a regular basketball court (hoop 3.05 m from ground) using a standard basketball (size 7 for males; size 6 for females). Players were instructed to make as many free-throws as possible, shooting within 5 s after receiving the ball as per international regulations and used previously in basketball research (Filipas et al., 2021). However, players normally completed this task within 1 min to not take up a considerable portion of end-of-quarter breaks. The overall number of free-throws made out of the 100 attempts across the entire simulation protocol (4 quarters × 25 shots) was recorded. No encouragement or feedback were provided throughout the shooting task.

#### 2.2.4 Perceptual-physiological variables

Heart rate (HR) was continuously monitored throughout the simulation protocol using Firstbeat Sports sensors, which have supported reliability and validity (Bogdány et al., 2016). Mean and peak absolute HR (beats·min^−1^) recorded for each player when completing the circuits (i.e., excluding breaks) in the simulation protocol were considered for reliability analyses. Mean and peak relative HR (%HR_max_) attained during the simulation protocol were determined for discriminative validity analyses calculated relative to age-predicted maximum HR (i.e., 220 – age in years). Blood lactate concentration (BLa) was measured via capillary samples taken from the earlobe immediately after the competition of the final circuit in each quarter using a portable amperometric lactate analyzer with supported validity and reliability (Crotty et al., 2021) (Lactate Pro 2, Arkray, Kyoto, Japan). The average BLa determined across all quarters was used for statistical analyses. Rating of perceived exertion (RPE) was collected 30 min after the completion of the entire simulation protocol using the validated (Chen et al., 2002) Borg’s Category Ratio-10 scale (Borg, 1998) used widely in basketball research (Ferioli et al., 2021 Kamarauskas et al., 2024).

### 2.3 Statistical analysis

All variables (except distance covered) were shown to be normally distributed with Shapiro–Wilk tests and are reported as mean ± standard deviation (SD) (with median and interquartile ranges also calculated for distance covered). Retest reliability was assessed via determination of coefficient of variation (CV) and intraclass correlation coefficient (ICC) statistics with 90% confidence intervals (CI) using customized spreadsheets (Hopkins, 2015) via the log-transformed variable. Discriminant validity analyses were performed by comparing outcomes between sexes and between playing levels using independent t-tests (while a non-parametric approach [Mann–Whitney *U* test] was applied to distance covered). Cohen’s *d* with 90% CI was calculated to indicate the magnitude of differences in pairwise comparisons for parametric data and was interpreted as follows: *trivial*, <0.20; *small*, 0.20–0.59; *moderate*, 0.60–1.19; *large*, 1.20–1.99; *very large*, ≥2.00 (Hopkins et al., 2009). The *r*-value, calculated as Z / SQRT(N) (Fritz et al., 2012), was determined as an effect size for pairwise comparisons in non-parametric data and interpreted using Cohen’s benchmarks as: *no effect*, <0.10; *small*, 0.10–0.29; *medium*, 0.30–0.49; and *large*, ≥0.50. Statistical significance was set at *p* < 0.05. Analyses were conducted using the *jamovi* package (The jamovi project, version 1.8^2^).

## 3 Results

### 3.1 Reliability analyses

Descriptive data for all variables across repeated trials and retest reliability statistics for the simulation protocol are provided in Table 1. Physical variables were mostly characterized by strong reliability statistics (ICC = 0.82–0.96; CV = 1.78–6.75%). However, decrement measures among the physical variables (ICC = 0.52–0.75; CV = 19.19–30.85%) and technical performance (free-throws made) (ICC = 0.73; CV = 10.54%) displayed weaker ICC and higher CV. Regarding perceptual-physiological variables, higher reliability was observed for HR variables (ICC = 0.72–0.78; CV = 1.82–2.16%), with lower reliability apparent for BLa and RPE (ICC = 0.53–0.69; CV = 10.34–20.96%).

**Table 1:** Physical, technical, and perceptual-physiological variables (mean ± standard deviation) measured during the game-specific basketball activity simulation protocol across repeated trials alongside reliability statistics.

Variable	Trial 1	Trial 2	ICC (90%CI)	CV% (90%CI)
**Physical variables**				
Movement load (AU)	199 ± 35	204 ± 24	0.82 (0.57; 0.93)	6.75 (5.01; 10.66)
Movement intensity (AU · min^−1^)	6.26 ± 1.04	6.37 ± 0.74	0.89 (0.72; 0.96)	5.02 (3.73; 7.88)
Mean sprint time (s)	1.48 ± 0.11	1.48 ± 0.11	0.91 (0.77; 0.97)	2.45 (1.83; 3.82)
Mean circuit time (s)	25.22 ± 2.12	24.78 ± 1.73	0.93 (0.81; 0.97)	2.28 (1.78; 3.56)
Mean jump height (cm)	33.27 ± 8.79	33.72 ± 7.85	0.96 (0.90; 0.99)	6.75 (5.00; 10.65)
Total distance covered (m)	4,427 ± 182	4,443 ± 218	0.89 (0.72; 0.96)	1.78 (1.33; 2.77)
Sprint time decrement (%)	11.03 ± 3.75	9.51 ± 2.07	0.62 (0.20; 0.84)	19.19 (14.35; 29.76)
Circuit time decrement (%)	8.54 ± 4.05	9.40 ± 5.12	0.75 (0.43; 0.90)	27.74 (20.74; 43.02)
Jump height decrement (%)	17.94 ± 8.07	17.47 ± 6.38	0.52 (0.06; 0.80)	30.85 (23.06; 47.83)
**Technical variable**				
Free-throws made (count)	71.3 ± 13.4	73.0 ± 11.2	0.73 (0.39; 0.89)	10.54 (7.78; 16.81)
**Perceptual-physiological variables**				
Mean heart rate (beats·min^−1^)	179 ± 8	178 ± 6	0.72 (0.38; 0.89)	2.16 (1.61; 3.38)
Peak heart rate (beats·min^−1^)	192 ± 8	189 ± 5	0.78 (0.48; 0.91)	1.82 (1.35; 2.85)
Blood lactate concentration (mmol · L^−1^)	8.79 ± 1.97	8.34 ± 2.74	0.53 (0.07; 0.80)	20.96 (15.29; 34.31)
Rating of perceived exertion (AU)	7.69 ± 0.94	7.70 ± 1.46	0.69 (0.32; 0.88)	10.34 (7.64; 16.48)

AU, arbitrary units; CI, confidence intervals; CV%, coefficient of variation as a percentage; ICC, intraclass correlation coefficient.

### 3.2 Discriminant validity analyses between sexes

Descriptive data for all variables according to sex along with comparison statistics are presented in Table 2. Among the physical variables, males had significantly higher movement loads and intensities (*p* < 0.05, *moderate* effects), as well as superior mean sprint time and jump height compared to females (*p* < 0.05, *moderate*-to-*large* effects). In contrast, non-significant differences were apparent between sexes for mean circuit time, distance covered, and performance decrements (*p* > 0.05, *trivial*-to-*moderate* effects). Regarding technical performance, a non-significant (*p* > 0.05), *trivial* difference between sexes was apparent for free-throws made. Likewise, all perceptual-physiological variables were similar between sexes (*p* > 0.05, *small* effects), except for RPE, which was significantly higher in females than males (*p* < 0.05, *moderate* effect).

**Table 2:** Physical, technical, and perceptual-physiological variables (mean ± standard deviation) alongside comparison statistics for discriminant validity analyses between sexes and playing levels during the game-specific basketball activity simulation protocol.

Variable	Competitive male	Recreational male	Competitive female	Sex comparison			Playing level comparison		
				*p*-value	ES (90%CI)	Interpretation	*p-*value	ES (90%CI)	Interpretation
Sample size (n)	13	13	9						
**Physical variables**									
Movement load (AU)	215 ± 31	186 ± 28	187 ± 28	**0.041**	0.97 (0.15; 1.75)	*Moderate*	**0.018**	1.02 (0.25; 1.75)	*Moderate*
Movement intensity (AU · min^−1^)	6.73 ± 0.96	5.80 ± 0.86	5.84 ± 0.87	**0.040**	0.97 (0.15; 1.75)	*Moderate*	**0.018**	1.02 (0.26; 1.75)	*Moderate*
Mean sprint time (s)	1.45 ± 0.10	1.48 ± 0.18	1.58 ± 0.13	**0.016**	−1.14 (−1.93; −0.31)	*Moderate*	0.618	−0.20 (−0.84; 0.46)	*Trivial*
Mean circuit time (s)	24.4 ± 2.0	26.3 ± 2.0	24.5 ± 1.0	0.866	−0.07 (−0.79; 0.64)	*Trivial*	**0.019**	−0.99 (−1.69; −0.24)	*Moderate*
Mean jump height (cm)	35.0 ± 5.8	–	22.8 ± 7.3	**<0.001**	1.92 (0.91; 2.86)	*Large*	–	–	–
Total distance covered (m)	4,479 ± 93*	4,214 ± 295	4,418 ± 273*	1.000	0.00	*Trivial*	**0.002**	0.57	*Large*
Sprint time decrement (%)	10.9 ± 3.9	10.8 ± 3.1	12.5 ± 5.3	0.837	−0.14 (−1.19; 0.92)	*Trivial*	0.953	0.02 (−0.62; 0.67)	*Trivial*
Circuit time decrement (%)	8.1 ± 3.4	12.2 ± 4.9	9.8 ± 4.1	0.303	−0.46 (−1.18; 0.28)	*Small*	**0.020**	−0.98 (−1.68; −0.24)	*Moderate*
Jump height decrement (%)	17.2 ± 5.2	–	21.8 ± 9.2	0.184	−0.64 (−1.43; 0.17)	*Moderate*	–	–	–
**Technical variable**									
Free-throws made (count)	66.2 ± 14.3	61.5 ± 18.4	68.9 ± 15.3	0.681	−0.18 (−0.89; 0.54)	*Trivial*	0.467	0.29 (−0.37; 0.94)	*Small*
**Perceptual-physiological variables**									
Mean heart rate (%HR_max_)	91.8 ± 2.3	89.0 ± 3.6	90.2 ± 4.3	0.266	0.50 (−0.24; 1.22)	*Small*	**0.023**	0.95 (0.22; 1.66)	*Moderate*
Peak heart rate (%HR_max_)	97.8 ± 2.3	95.9 ± 3.0	96.2 ± 3.5	0.197	0.58 (−0.17; 1.31)	*Small*	0.078	0.72 (0.02; 1.40)	*Moderate*
Blood lactate concentration (mmol · L^−1^)	9.12 ± 2.59	8.52 ± 2.46	8.13 ± 2.44	0.382	0.39 (−0.35; 1.10)	*Small*	0.551	0.23 (−0.42; 0.88)	*Small*
Rating of perceived exertion (AU)	7.14 ± 0.95	8.34 ± 1.25	8.11 ± 0.60	**0.011**	−1.08 (−1.80; −0.32)	*Moderate*	**0.011**	−1.08 (−1.80; −0.32)	*Moderate*

AU, arbitrary units; CI, confidence intervals; ES, effect size. Bolded *p*-value indicates statistically significant difference at *p* < 0.05; * indicates data were not normally distributed (median ± inter-quartile range: competitive males = 4,519 ± 0 m; competitive females = 4,519 ± 0 m), so pairwise comparisons were performed with the Mann Whiteney *U* test and *r*-value effect size interpreted according to Cohen’s benchmarks; − indicates no jump data for recreational males given it were not collected due to technical issues.

### 3.3 Discriminant validity analyses between playing levels

Descriptive data for all variables according to playing level along with comparison statistics are also presented in Table 2. Regarding physical variables, competitive males had significantly higher movement loads, movement intensities, and total distances, alongside significantly superior mean circuit time and circuit time decrement than recreational males (*p* < 0.05, *moderate*-to-*large* effects). In turn, comparable mean sprint time and sprint time decrement were evident between playing levels (*p* > 0.05, *trivial* effects). Likewise, a non-significant (*p* > 0.05), *trivial* difference in free-throws made was evident between playing levels. Among the perceptual-physiological variables, competitive males had significantly higher mean HR and lower RPE (*p* < 0.05, *moderate* effects), but non-significantly higher peak HR and BLa responses (*p* > 0.05, *small*-to-*moderate* effects) compared to recreational males.

## 4 Discussion

In addressing the first aim, this study outlines a new basketball activity simulation protocol that is more representative of typical playing durations experienced during games than the original simulation protocol proposed in the literature (Scanlan et al., 2012, 2014). In turn, we also aimed to examine the reliability and discriminant validity of this new simulation protocol to inform end-users on its potential utility in practice. Our findings revealed that physical and HR variables demonstrated relatively strong reliability, while physical decrement, technical, and other perceptual-physiological variables displayed weaker reliability. Discriminant validity of the protocol was also demonstrated via differences in many variables emerging between players of different sexes and playing levels.

### 4.1 Reliability analyses

Regarding reliability analyses, unfounded statistical criteria are regularly referenced in the sport science literature to determine whether a testing protocol is reliable (Atkinson and Nevill, 1998). However, it is ultimately up to the end-user and their analytical goals to decide on the level of measurement error (i.e., reliability) they are willing to accept in their specific context when adopting a testing protocol in practice (Atkinson and Nevill, 1998). In this way, if test outcomes cannot be reliably reproduced, it cannot be effectively determined whether players have improved (Weakley et al., 2023). Accordingly, we provide some initial insight into the relative (via ICC) and absolute (via CV) reliability of several variables within physical, technical, and perceptual-physiological domains during the game-specific simulated basketball activity protocol to help guide decision-making among end-users. More precisely, most physical variables (except decrement measures) had the strongest relative reliability (ICC ≥0.82), meaning they may be most useful for discriminating between players (e.g., assessing player rankings within the team across time) (Impellizzeri and Marcora, 2009). Likewise, several physical variables (CV <7%) and both HR variables (CV ~2%) displayed the strongest absolute reliability, indicating they may hold most utility in longitudinal assessments (e.g., assessing changes in response to interventions or across seasonal phases) (Impellizzeri and Marcora, 2009). In contrast, we observed physical decrement, technical, and remaining perceptual-physiological variables to be least reliable with ICC of 0.52–0.75 and CV of 10–31%.

The reliability statistics we reported are generally weaker than those documented for the original version of the simulation protocol (ICC = 0.56–0.99, CV = 1–17%) (Scanlan et al., 2014). This variation in reliability statistics across studies may be due various factors such as the higher training status (i.e., regional and semi-professional levels) of the players recruited previously promoting more consistent performances than players in our study, as well as the different technologies used for measurements (e.g., timing sprint and circuit times, monitoring movement load). Nevertheless, the trends in reliability statistics we observed align with those reported for the original version of the simulation protocol (Scanlan et al., 2014), as well as other simulated team sport activity protocols in soccer (Williams et al., 2010) and rugby league (Waldron et al., 2013). Specifically, it was reported that physical and HR variables tended to have the strongest reliability and physical decrement (Scanlan et al., 2014), technical (Williams et al., 2010), BLa (Waldron et al., 2013), and RPE (Williams et al., 2010) variables had the weakest reliability. Indeed, physical decrement variables have been shown to possess considerably low reliability during repeated-sprint and multi-dimensional movement tasks systemically across the literature among soccer players, possibly due to permutations in pacing strategies adopted (Altmann et al., 2019). Consequently, the collective evidence indicates that the variations in reliability we observed across different types of variables may be rather universal in team sport simulation protocols.

### 4.2 Discriminative validity analyses

To assess discriminant validity, we examined differences in variables taken during the simulation protocol between player samples of different sexes (comparable playing levels) and playing levels (males). Regarding sex comparisons, some variables were significantly different between males and females, with males demonstrating greater physical outputs (i.e., movement load, movement intensity, mean sprint time, and mean jump height) but lower RPE than females. The superior physical intensities among males may be expected given the players were competing at comparable playing levels and completing similar team training routines. In this regard, adult males have been documented to possess greater strength, power, and speed attributes than adult females of similar age and training status (Hunter et al., 2023), which contribute strongly to several physical variables we measured. In contrast, other variables, such as physical decrements, total distance, physiological intensities, and shooting performance, were rather comparable between sexes. These findings may be expected given the greater physical outputs demonstrated by males may be countered by superior performance fatiguability in females (Hunter, 2016) to promote consistent decrement and total distance outcomes between sexes. Moreover, maximal HR responses during exercise and sporting technical skills have been suggested to display minimal sex differences (Hunter et al., 2023). Consequently, the lack of differences between sexes among some variables may not diminish the discriminant validity of the simulation protocol.

Regarding playing level comparisons, we would expect players undertaking structured team training and game schedules in competitive environments to possess superior performance during the simulation protocol than those participating in recreational settings. In this regard, competitive players displayed a significantly higher movement load, movement intensity, and total distance, alongside faster circuit times and decrements than recreational players. Given anaerobic and aerobic fitness attributes have been shown to significantly correlate with physical variables during the original version of the simulation protocol (Scanlan et al., 2014), the higher training demands, and therefore likely higher fitness status, of the competitive players may underpin these differences. In support of this notion, male basketball players competing at higher playing levels within Italian competitions (ranging from amateur to professional) have been shown to possess superior fitness across a range of anaerobic and aerobic attributes than lower-level players (Ferioli et al., 2018). We also observed competitive players to maintain significantly higher mean relative HR across the simulation protocol with lower RPE than recreational players. These variations suggest competitive players were able to maintain higher cardiovascular intensities in completing the set activities with less perceptual stress, which may also be attributed to them possessing a greater aerobic fitness, especially given the strong oxidative metabolic contribution involved in completing the simulation protocol (Latzel et al., 2018). In contrast, non-significant differences were evident for sprint variables, as well as shooting performance, between playing levels. Similarities in these measures may be anticipated given sprint performance times over short distances have been shown to vary and potentially overlap across a range of playing levels ranging from amateur to professional in a systematic review encompassing sprint testing outcomes in male basketball players (Morrison et al., 2022). Moreover, while shooting performance may typically be better in players participating in more elite competitions (Zuzik, 2011), the technical shooting abilities of competitive players in our study may have been more closely matched with the recreational players, especially for common, standardized tasks such as the free-throw. In support of this notion, non-significant differences have also been observed in free-throw shooting performance across similar shooting protocols between intermediate-level competitive and novice, male basketball players (Pakosz et al., 2021).

### 4.3 Limitations

In interpreting our findings, the limitations encountered should be considered. Firstly, we examined only one modified version of the simulation protocol, equating to a playing time of 32 min interspersed with 31 min of passive recovery. Accordingly, this protocol may not be practically applicable to player samples who experience alternative exposures during games. Secondly, the original version of the simulation protocol was developed using video-based time-motion data from professional, male players (Scanlan et al., 2012, 2014); however, we examined competitive players competing at lower levels than this as well as recreational players. Consequently, the demands elicited in our modified simulation protocol may not represent the precise activity profiles of games encountered among the players we recruited. Thirdly, while the Witty gate photocells (Stojanović et al., 2019; Gonzalo-Skok and Biship, 2024) and Firstbeat Sports technology sensors (Portes et al., 2022, 2023) have been previously used to assess physical demands in basketball players, the precise validity and reliability of these devices are yet to be investigated. Consequently, interpretation of the physical demand data reported in our study should be conducted in consideration of this point. Fourthly, we focused on examining discriminant validity given its importance in applied sport science contexts for distinguishing between different player samples. However, other forms of validity are also important for application in practice (e.g., criterion validity, ecological validity) (Weakley et al., 2023) and warrant further investigation. Finally, we could not recruit sufficient players to perform reliability analyses according to player sex and playing level nor conduct more detailed discriminant analyses via comparisons between sexes within each playing level or between playing levels within each sex. Therefore, similar research on this topic is encouraged across wider samples of male and female players competing at various playing levels.

## 5 Practical applications and conclusions

The predominant practical outcome from this study is the development of a new basketball activity simulation protocol that is more specific in replicating actual playing durations and game configurations than the original version (Scanlan et al., 2012). Consequently, the stimuli elicited and insight gathered from this new simulation protocol likely hold stronger translation to real competitive contexts. For instance, the simulation protocol could be used to assess the efficacy of different interventions (e.g., nutritional supplementation, training strategies) (Delextrat et al., 2018; Hovsepian et al., 2021) or compromised conditions (e.g., mental fatigue, sleep restriction) (Bourdas et al., 2024) on game-specific basketball activity capabilities. Accordingly, the new simulation protocol may provide researchers and practitioners with more holistic information compared to classic physical fitness tests such as jump, sprint, or change-of-direction assessments that are restricted to a specific form of activity. Furthermore, the simulation protocol could also be applied as a training tool (Javanmardi et al., 2021) in practice. In turn, we also provide useful reliability data for physical, technical, and perceptual-physiological variables measured during the simulation protocol to inform end-users on the inherent measurement error that may be encountered. In this regard, most physical variables and HR variables displayed the strongest reliability; however, caution should be exercised in interpreting performance decrement variables in particular given the relatively weak reliability observed for them, which is in line with other research findings (Scanlan et al., 2014; Altmann et al., 2019). We also provide support for the simulation protocol in detecting differences in selected variables between sexes and playing levels that may be expected to vary based on these factors. In this regard, the simulation protocol may hold utility in benchmarking or selecting basketball players as part of team processes.

## Data availability statement

The raw data supporting the conclusions of this article will be made available by the authors, without undue reservation.

## Ethics statement

The studies involving humans were approved by UCAM Universidad Católica de Murcia’s Ethics Committee. The studies were conducted in accordance with the local legislation and institutional requirements. The participants provided their written informed consent to participate in this study.

## Author contributions

DF: Conceptualization, Data curation, Formal analysis, Investigation, Methodology, Project administration, Supervision, Writing – original draft. PA: Writing – review & editing, Funding acquisition, Methodology, Resources. TF: Methodology, Writing – review & editing. FT: Writing – review & editing. DC: Formal analysis, Writing – review & editing. LF: Investigation, Writing – review & editing. LC: Investigation, Methodology, Writing – review & editing. AS: Visualization, Writing – original draft.

## Footnotes

^1^ https://www.acb.com/estadisticas-individuales/minutos

^2^Retrieved from: https://www.jamovi.org.
